# Supplementary material for: Chiral nanocrystals grown from MoS2 nanosheets enable photothermally modulated enantioselective release of antimicrobial drugs
Source: Nat Commun. 2022 Nov 26;13:7289. doi: 10.1038/s41467-022-35016-8 (PMC9701227; doi:10.1038/s41467-022-35016-8)
Supplement: Supplementary file 1 — Supplementary Figures and Notes [file 41467_2022_35016_MOESM1_ESM.pdf]

Supplementary Information

Chiral nanocrystals grown from MoS_2 nanosheets enable photothermally modulated enantioselective release of antimicrobial drugs

Bang Lin Li,[1,*] Jun Jiang Luo,[1] Hao Lin Zou,[1] Qing-Meng Zhang,[1] Liu-Bin Zhao,[1] Hang Qian,[2] Hong Qun Luo,[1] David Tai Leong,[3,*] & Nian Bing Li[1,*]

[1] Key Laboratory of Luminescence Analysis and Molecular Sensing, Ministry of Education, School of Chemistry and Chemical Engineering, Southwest University, Chongqing 400715, P. R. China

[2] Institute of Respiratory Diseases, Xinqiao Hospital, Third Military Medical University, 183 Xinqiao Street, Chongqing, 400037, P. R. China.

[3] Department of Chemical and Biomolecular Engineering, National University of Singapore, Singapore 117585, Singapore.

*Correspondence and requests for materials should be addressed to B. L. Li (chemlibl@swu.edu.cn), D. T. Leong (cheltwd@nus.edu.sg), or N. B. Li (linb@swu.edu.cn).

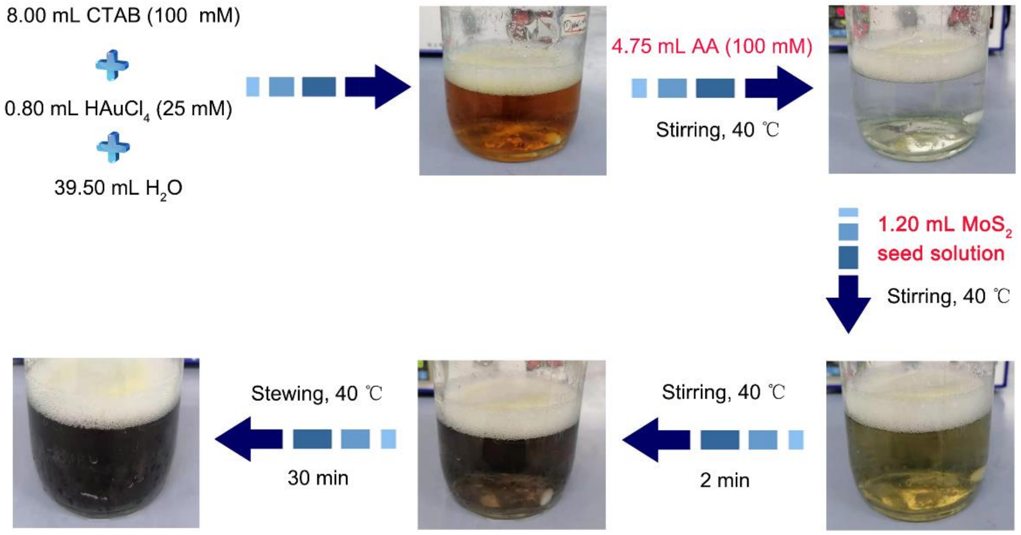

Supplementary Fig. 1 Reaction between $HAuCl_4$ and MoS_2 nanosheets. The schematic illustration showing the reaction processes of $HAuCl_4$ and exfoliated MoS_2 in the presence of the hexadecyl trimethyl ammonium bromide (CTAB) and ascorbic acid (AA).

Supplementary Note 1. Synthesis process of nanocrystals

The yellow-color solution is formed once the $HAuCl_4$ solution is added into the CTAB dispersion, and the conjugated CTAB-Au^{3+} is constructed (Supplementary Fig. 1). After that, the reductant, AA, can reduce CTAB-Au^{3+} conjugated compounds to CTAB-Au^{+}.[1] The mixture turned into the colorless. In this step, none of plasmonic Au nanostructures was obtained. The addition of green-color MoS_2 seed solution lead to the immediate alternation of mixture colors, which trended to be stable dispersion after 30 min at 40 ℃. It is supposed that MoS_2 can offer both electrons and substrates for the sequential growth of Au nanostructures on the heterostructural surface. The growth of Au nanostructures is highly modulated by the MoS_2 and co-existing small molecules, and this point is demonstrated in the further works.

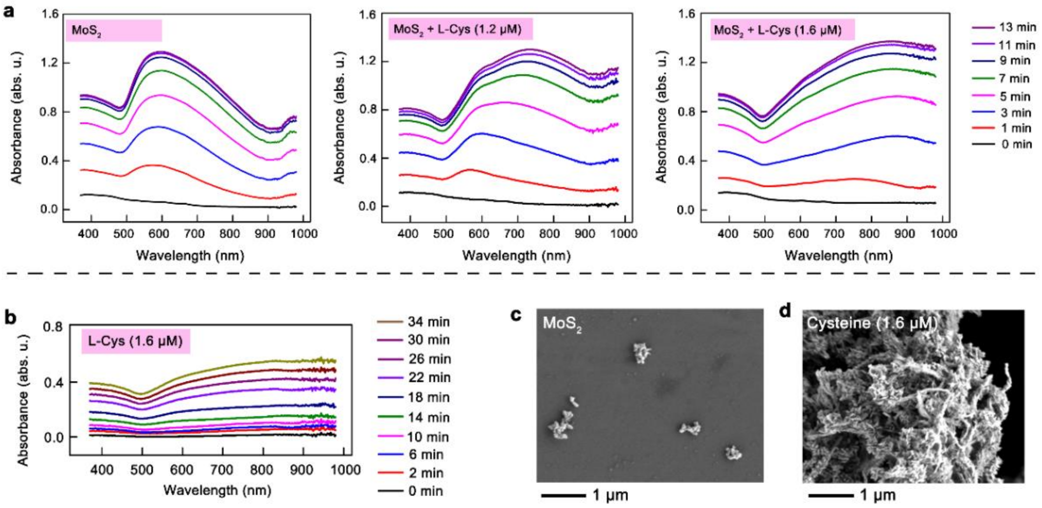

Supplementary Fig. 2 Spectroscopic and scanning electron microscopy records of nanocrystal products in diverse reaction. **a** Time-resolved optical absorption spectra reflecting the reaction processes between exfoliated MoS_2 nanosheets and CTAB-Au^+ in the absence and presence of 1.2 and 1.6 μM L-Cys. **b** Time-resolved optical absorption spectra of the CTAB-Au^+ solution with the addition of 1.6 μM L-Cys. **c**, **d** SEM images of CTAB-Au^+ reaction products with the addition of exfoliated MoS_2 (**c**) and 1.6 μM L-Cys (**d**), respectively. **a**, **b** Source data are provided in Source Data file.

Supplementary Note 2. Time-resolved optical characterization and additional scanning electron microscopy results

The time-resolved vis-NIR absorption spectra indicated that the reactions CTAB-Au^+ and MoS_2 terminated after 13 min (Supplementary Fig. 2a). In the absence of L-Cys, the absorption peak always located at around 580 nm during the whole reaction process. However, with the addition of L-Cys (1.2 μM), the absorption peaks shifted from about 570 nm to 750 nm when the reaction times increased from 1 min to 13 min. Therefore, it was supposed that before the formation of dendritic nanostructures, the Au/MoS_2 hybrid seeds were firstly constructed. In the presence of 1.6 μM L-Cys, the absorption peaks are found at wavelength of 760 nm within 1 min, suggesting the rapid reaction involved by L-Cys. Finally, the reaction solution peaked at about 870 nm. The high concentration (1.6 μM) of L-Cys causes the difficult in capturing the transition morphologies. As a control experiment, the reaction between CTAB-Au^+ and L-Cys (1.6 μM) was explored. The reaction time largely extended, and only a wide adsorption range was found. SEM suggests that in the absence of MoS_2, cysteine induces the formation of nanowires, rather than size-confined nanocrystals (Supplementary Fig. 2c and d).

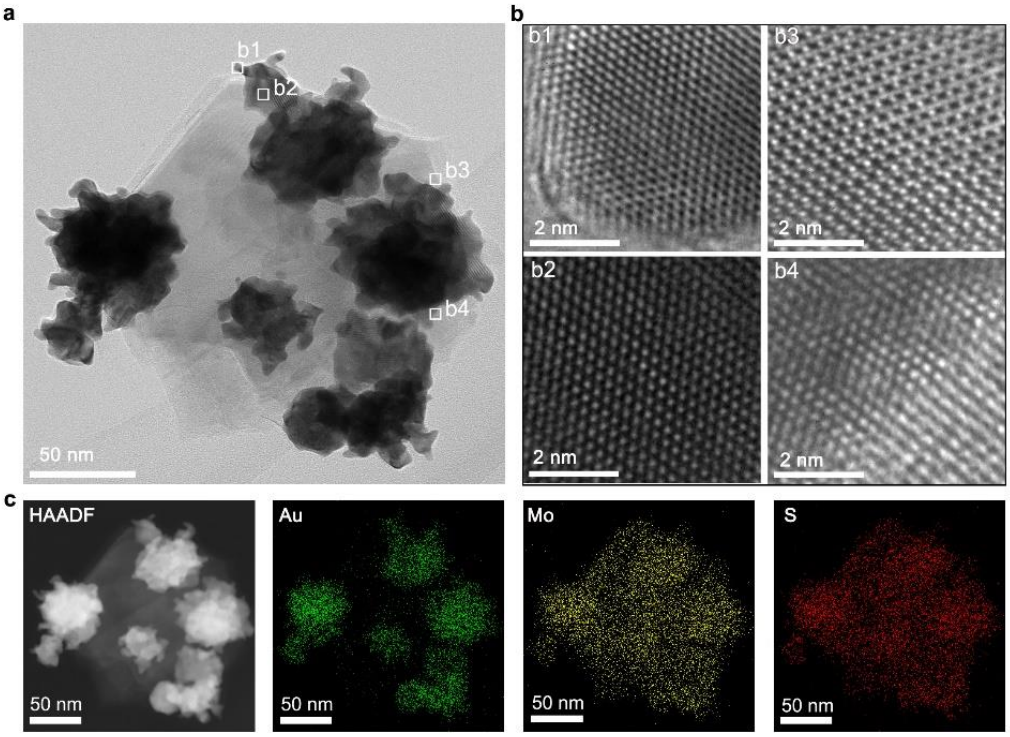

Supplementary Fig. 3 Characterization of nanocrystals synthesized from the large-size MoS_2 nanosheets. a-c (**a**) Transmission electron microscope (TEM), (**b**) high-resolution TEM images (HRTEM), (**c**) high-angle annular dark field (HAADF) and energy-dispersive X-ray spectra (EDX) element scan images of branched nanocrystals, synthesized from the addition of MoS_2 layers and 1.2 μM L-Cys.

Supplementary Note 3. Structural characterization of nanocrystals

When the larger-size MoS_2 nanosheets were adopted for directing the growth of Au/MoS_2 nanostructures, the products were collected and characterized. From the TEM image, it is shown that flower-shaped nanostructures are supported on a large plane (Supplementary Fig. 3a). The composition and morphologies are further analyzed via the HRTEM, HAADF, and EDX results (Supplementary Fig. 3b). The plane belongs to the semiconducting MoS_2, whereas the flower nanostructures are identified as the Au/MoS_2 nano-heterostructures. Overall, the size of MoS_2 nanosheets will influence the formation of Au/MoS_2 nanostructures. As is shown in Supplementary Fig. 3, when the plane size of MoS_2 is larger than 200 nm, the Au-covering nanostructures could not be obtained. The self-sacrificed models which use MoS_2 as the growing substrate were not suitable for all sizes of MoS_2.

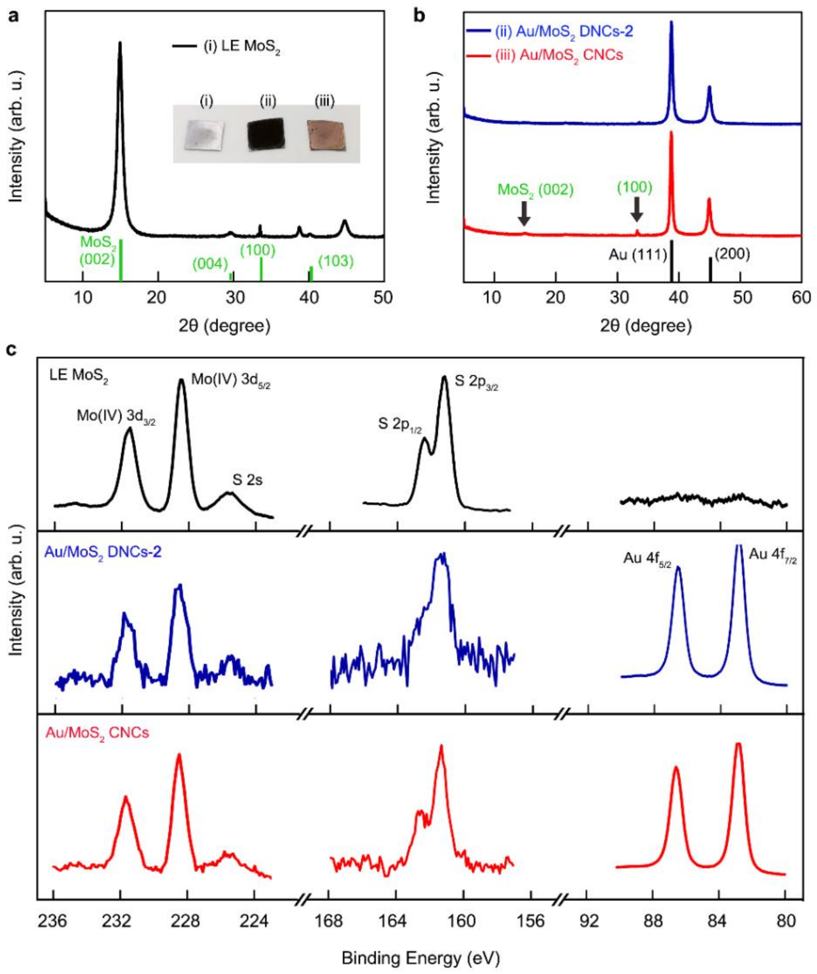

Supplementary Fig. 4 Characterizations of diverse Au/MoS_2 products. (**a** and **b**) The X-ray diffraction (XRD), photos of the nanomaterials-covering silicon chips (inset pictures in supplementary Fig. 3a). (**c**) X-ray photoelectron spectroscopy (XPS) results of liquid-exfoliated (LE) MoS_2, CNCs, and DNCs-2. Source data are provided in Source Data file.

Supplementary Note 4. X-ray diffraction and X-ray photoelectron spectroscopy measurements

The XRD characteristic peaks corresponding to the (002) and (100) crystal planes of semiconducting MoS_2 were found in the LE MoS_2 sample (Supplementary Fig. 4a and b). In terms of Au/MoS_2 CNCs, the slight characteristic peaks of MoS_2 and strong characteristics peaks of Au were displayed, which might be attributed to the coverage of Au on MoS_2. In comparison to CNCs, the DNCs-2 exhibit less XRD signals of MoS_2. The cracked structures of CNCs are supported. The results were also demonstrated by the XPS measurements, indicating the composition of Au and MoS_2 in CNCs and DNCs-2 (Supplementary Fig. 4c).[2]

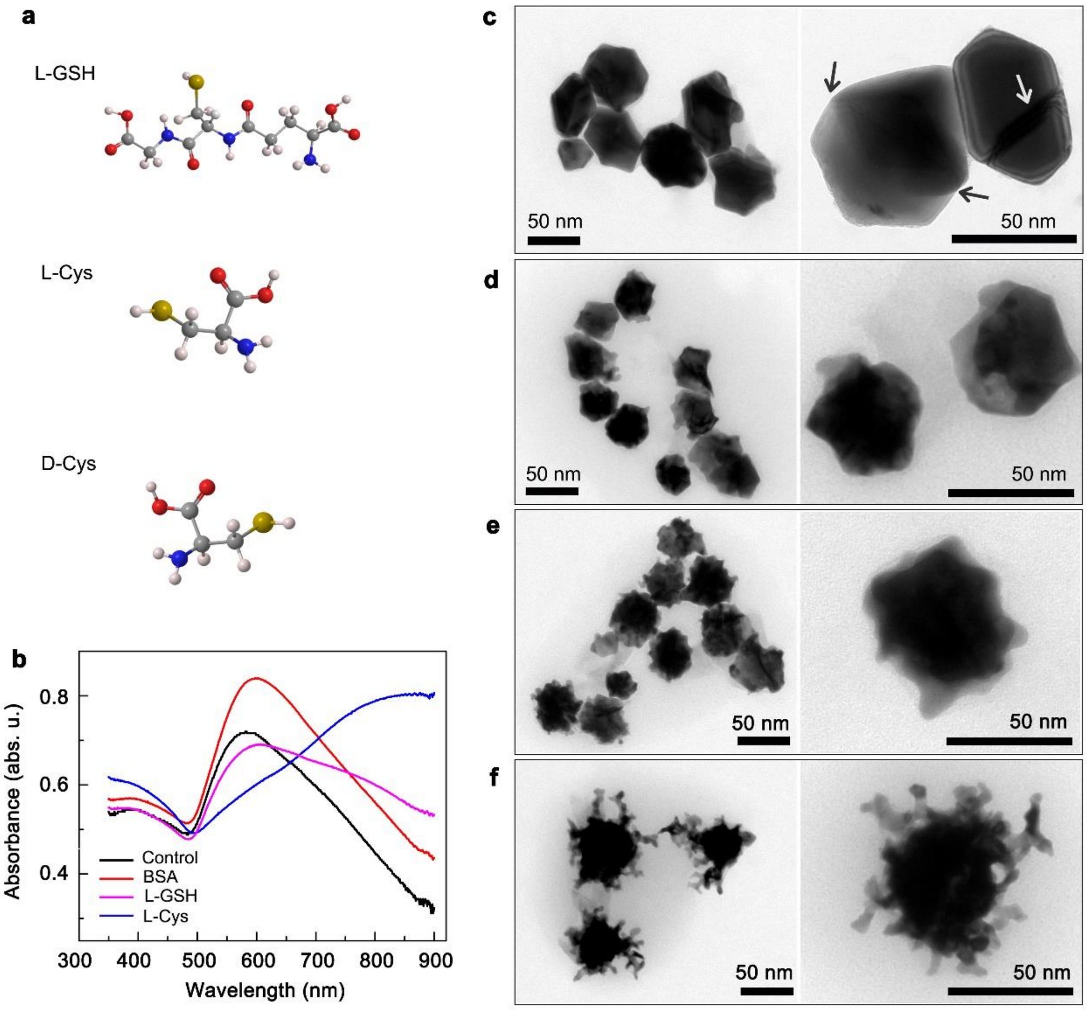

Supplementary Fig. 5 Preparation of branched nanocrystals at MoS_2 interfaces in the absence and presence of different thiol compounds. a Schematic illustration of L-glutathione (L-GSH), L-Cys, and D-Cys with different molecular configuration. **b** Absorption spectra of MoS_2-endowed Au nanostructures in the presence of different molecules. BSA: 2.0 mg mL^{-1}; L-GSH and L-Cys: 1.6 μM. Source data are provided in Source Data file. **c-f** TEM images of Au/MoS_2 nanoheterostructures prepared in the absence (**c**) and presence of (**d**) BSA, (**e**) L-GSH, and (**f**) L-Cys. The scale bars are 50 nm.

Supplementary Note 5. Optical and morphological characterizations of nanocrystals synthesized from other thiol compounds

Instead of cysteine, other thiol compounds, such as bovine serum albumin (BSA), and L-glutathione (L-GSH), were used for the synthesis of Au/MoS_2 nano-heterostructures. In the presence of BSA and L-GSH, the branched structures were observed, which were lower-level than that synthesized from cysteine. The conclusion was consistently supported by the absorption spectra and TEM images (Supplementary Fig. 5).

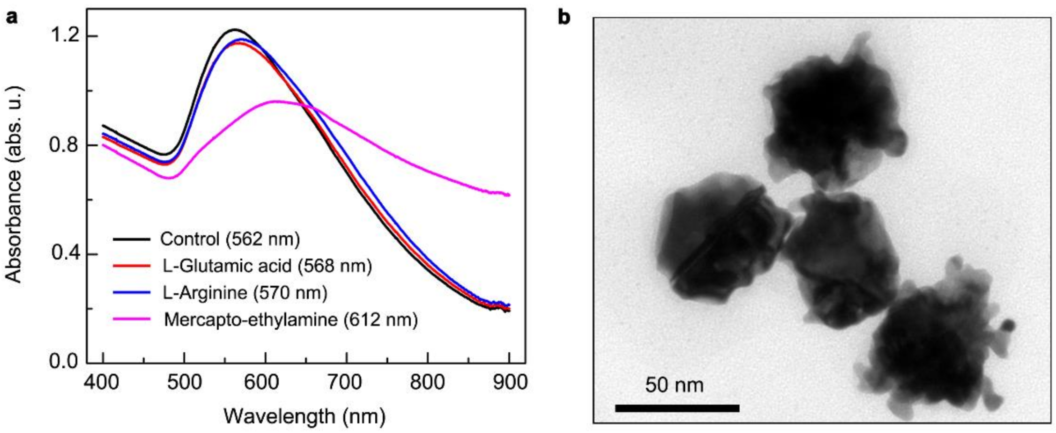

Supplementary Fig. 6 Comparison of Au/MoS$_2$ products in the presence of different molecules. a Absorption spectra of Au/MoS$_2$ nano-heterostructures in the absence (Control sample) and presence of L-glutamic acid, L-arginine, and mercapto-ethylamine. The concentrations of compounds are 1.6 μM. Source data are provided in Source Data file. **b** TEM image of Au/MoS$_2$ nanoheterostructures in the presence of mercapto-ethylamine.

Supplementary Note 6. Comparison of nanocrystals synthesized from compounds without and with thiol groups

The absorption spectra of nanostructures prepared from the L-glutamic acid, and L-arginine display the absorption peaks consistent with that in the absence of amino acids (Supplementary Fig. 6a). Nevertheless, when the mercapto-ethylamine was adopted, a large red-shift of peak was found on its absorption spectrum, altering from 562 to 612 nm. In order to identify the red shift, the TEM image of nanostructures derived from the mercapto-ethylamine was shown (Supplementary Fig. 6b). Obviously, slight branched structures can also be noticed in the mercapto-thylamine sample.

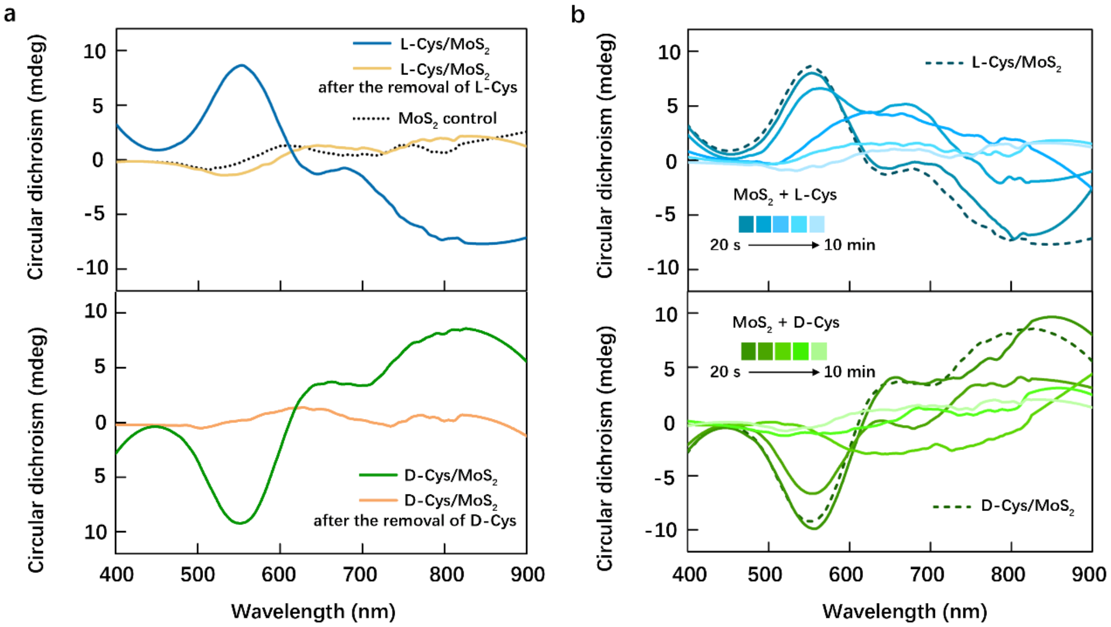

Supplementary Fig. 7 Synthesis of chiral nanocrystals under different conditions. a Circular dichroism (CD) spectra of as-synthesized Au/MoS_2 hybrids before and after the free cysteine molecules (L-Cys and D-Cys) in the solutions have been removed *via* the centrifugation route. The initial concentrations of L-Cys and D-Cys are 1.2 μM, respectively. **b** CD spectra of as-synthesized Au/MoS_2 hybrids in the presence of L-Cys and D-Cys, respectively. The addition of 1.2 μM cysteine (L-Cys or D-Cys) was conducted after the exfoliated MoS_2 was added to the growth solutions (CTAB-Au^+) for different reaction times (20 s, 1.0 min, 3.0 min, 5.0 min, 10.0 min). As control samples, L-Cys and D-Cys were mixed with MoS_2, and then the mixtures were added to the growth solutions (L-Cys/MoS_2 and D-Cys/MoS_2). Source data are provided in Source Data file.

Supplementary Note 7. Circular dichroism measurements of nanocrystals prepared from different synthesis conditions

After the incubation of cysteine with MoS_2, the samples were centrifuged and the sediments were collected. We used the sediments as the seed materials for the growth of Au/MoS_2 hybrids. It was found that none of CD responses are observed from the treated MoS_2 samples (Supplementary Fig. 7a). CD spectra indicate that after the removal of free cysteine in solution, a small number of L-/D-cysteine molecules physisorbed on MoS_2 had none of impacts on the contribution of chiral materials.[3] In order to support the chirality transfer mechanism, the synthesis routes were changed (Supplementary Fig. 7b). Firstly, exfoliated MoS_2 was added to the growth solution (CTAB-Au^+). After different reaction times (20 s – 10.0 min), the cysteine solutions containing different L/D-enantiomers were added to the reaction solutions, respectively, and CD spectra were recorded

to characterize the chirality of final products. When the reaction times increased from 20 s to 1 min, the CD curves remain constant but their peaks slightly shift to longer wavelengths. However, the CD peaks gradually drop after the reaction times increase to 1.0 min, and none of CD signals are observed after 5 min. Therefore, it was supposed that the chiral nano-heterostructures were synthesized based on the MoS_2 interfaces. After the MoS_2 nanosheets were totally covered by the Au atomic layers, the products cannot be served as the growth seeds for the sequential formation of chiral Au/MoS_2 nano-heterostructures.

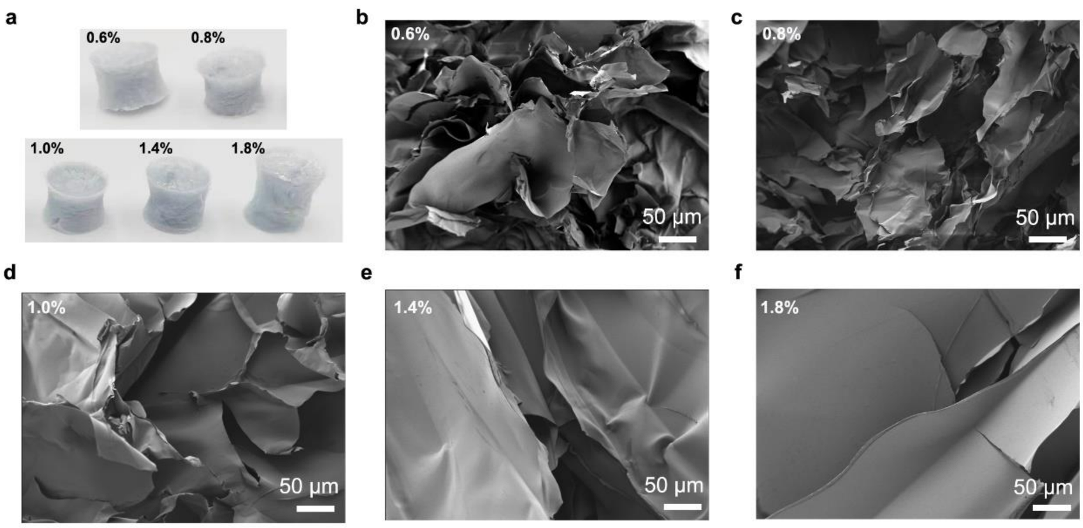

Supplementary Fig. 8 Agarose concentration-dependent hybrid hydrogels. The photos (**a**) and SEM images(**b-f**) of DNCs aerogels prepared from the different ratios of agarose. Scale bars in **b-f** are 50 μm.

Supplementary Note 8. Characterization of aerogels

The aerogels were prepared via the freezing-dry treatments of nanomaterials-doped agarose hydrogels. The amount ratios of agarose in the hydrogels altered from 0.6% to 1.8 %. From the real pictures, it was observed that the surfaces of aerogels became smoother with the increases of the amount of agarose, and the densities were also accordingly improved (Supplementary Fig. 8a). Obviously, the SEM images were recorded. Net structures of aerogels were found. A lot of holes with the sizes ranging from 100 to 2 μm were displayed as the increases of agarose ratios from 0.6% to 1.8% (Supplementary Fig. 8b-f). The holes in the aerogels and hydrogel permit the diffusion of small molecules in the aqueous and gas phases. Consequently, the aerogels and hydrogels can serve as the substrate used for the organic drug loading and releases.

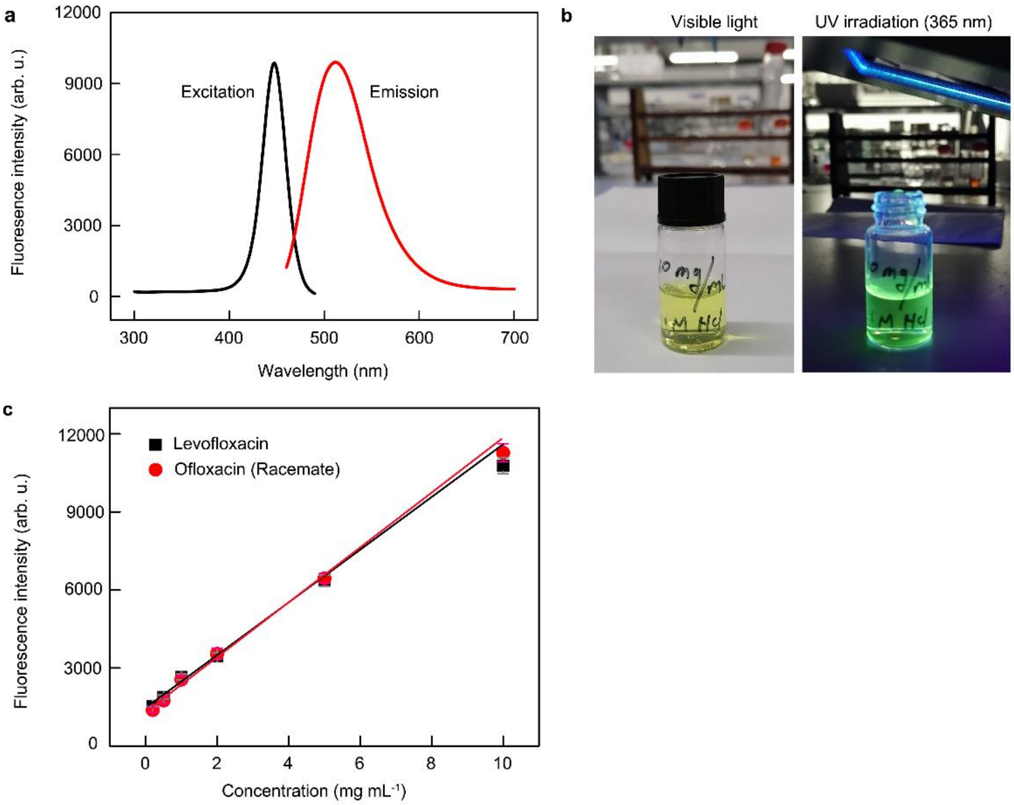

Supplementary Fig. 9 Fluorescence quantitative analysis of ofloxacin concentrations. a fluorescence excitation and emission spectra of levofloxacin (10.0 mg mL^{-1}) dispersed into the 1.0 M HCl solution. **b** Photos of the levofloxacin solution under the visible and UV (365 nm) light, respectively. **a**, **c** Source data are provided in Source Data file.

Supplementary Note 9. Fluorescence studies of ofloxacin solutions

From the fluorescence spectra of the levofloxacin solution (Supplementary Fig. 9a), the levofloxacin exhibits strong emission peaked at about 515 nm, and the peak of 445 nm is observed on the excitation spectrum. Meanwhile, the green fluorescence was noticed when the levofloxacin solution was irradiated under the UV light (Supplementary Fig. 9b). When the excitation wavelength is 445 nm, and the luminescence intensities are recorded at the wavelength of 515 nm. Significantly, the concentrations (C: mg mL^{-1}) of levofloxacin and the racemate of ofloxacin exhibit the consistent linear relationship to the fluorescence intensities (F), respectively (Supplementary Fig. 9c). The linear relationships are exhibited below: F = 1262.2 + 1030.7 C (levofloxacin), and F = 988.2 + 1350.3 C (the racemate), respectively. Therefore, the fluorescence intensities are used for the quantitative analysis of the concentrations of levofloxacin and the racemate of ofloxacin.

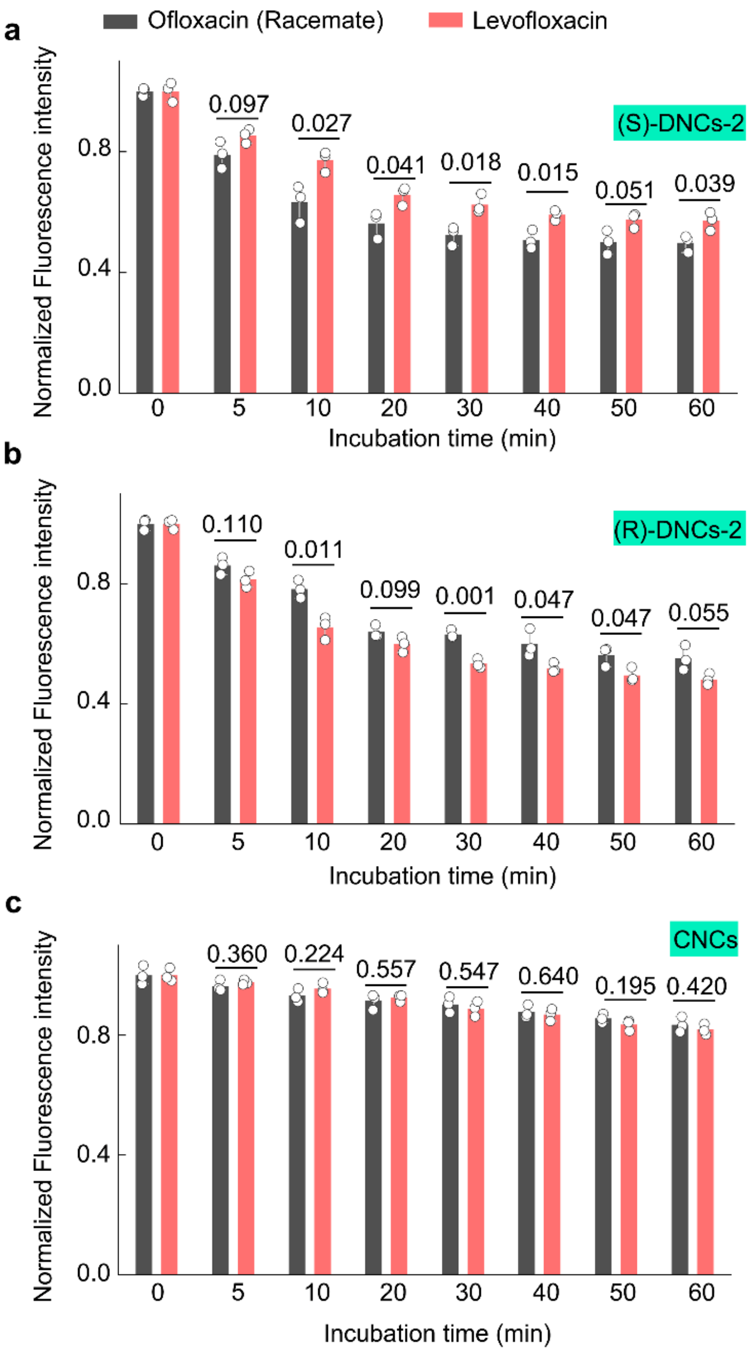

Supplementary Fig. 10 Discriminated adsorption performances of chiral Au/MoS_2 nanocrystals to enantiomeric ofloxacin molecules in aqueous solutions. a-c Normalized fluorescence intensities in the supernatant responding to the various incubation times of (S)-DNCs-2 (**a**), (R)-DNCs-2 (**b**) and CNCs (**c**) Au/MoS_2 nanostructures to ofloxacin racemate and levofloxacin, respectively. The concentrations of levofloxacin and ofloxacin are 10 μg mL^{-1}. The concentrations of nanostructures are 100 μg mL^{-1}. Error bars represent the standard deviation (SD) of independent experiments (n=3). One-sided tests were conducted and no adjustments were made for multiple comparison. Data are presented as mean values ± SD. Statistical analysis was performed through one-way ANOVA followed by students' t tests. Exact *P* values are inserted in the panels. Source data are provided in Source Data file.

Supplementary Note 10. Adsorption performance studies of chiral Au/MoS_2 nanocrystals to ofloxacin

Nanostructures were added to the ofloxacin solutions for incubation. After the mixtures were treated with diverse incubation times, the dispersions were centrifuged at a relative centrifugal force (10965 × g) for 2 min. The florescence in the supernatant was measured. With the increases of incubation times, the normalized fluorescence intensities correspondingly drop. In terms of (S)-DNCs-2 samples, after 60 min incubation, the normalized fluorescence intensity of ofloxacin racemate decline to 0.50, which is larger than that of levofloxacin (around 0.57). Nevertheless, contrast fluorescence change regulation of ofloxacin racemate and levofloxacin can be found on (R)-DNCs-2 samples. The value of ofloxacin racemate decreases to 0.55, but it largely declined to 0.48 in levofloxacin. In CNCs samples, the normalized fluorescence intensities of ofloxacin racemate and levofloxacin only declined to 0.83 and 0.82, respectively, indicating that CNCs nanostructures without chiral characteristics exhibit none of fluorescence decreases performances to ofloxacin enantiomers. The contrast fluorescence phenomenon in the aqueous solutions is attributed to the enantiomeric recognition and discriminate adsorption of ofloxacin enantiomers on chiral (R)/(S)-DNCs-2 nanostructures.

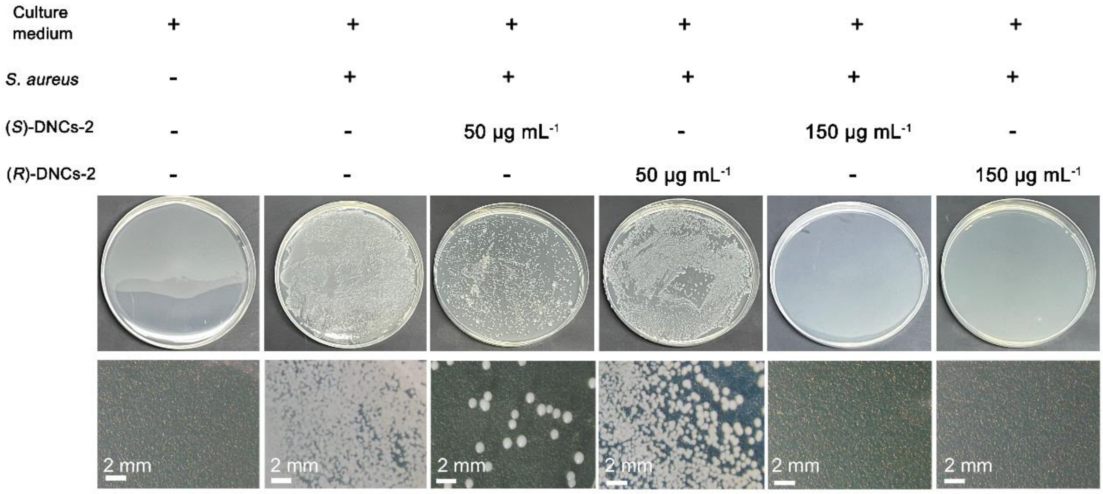

Supplementary Fig. 11 Antimicrobial studies of Au/MoS_2 nanocrystals using the co-incubation route. The photos (the top lane) and microscopic images (the bottom lane) of biological samples incubated for 24 h (37 ℃) in the absence and presence of nanomaterials. The bacteria culture was based on the co-incubation strategy. Scale bars shown in the microscopic images are 2 mm.

Supplementary Note 11. Antimicrobial performance studies of nanocrystals

In terms of the control sample, none of the *S. aureus* colonies was found (Supplementary Fig. 11), whereas the bacteria were full of the culture substrate when the S. Aureus was added on the culture medium. After the bacteria were incubated with DNCs-2 for 12 h, the culture of bacteria was conducted. Nevertheless, the number of colonies was largely decreased for both (*S*)-DNCs-2 and (*R*)-DNCs-2. Once the concentrations of nanomaterials increased to 150 μg mL^{-1}, there is none of *S. aureus* colonies being observed. The biological experiments results indicated that both the (*S*)-enantiomer and (*R*)-enantiomer of dendritic nanostructures exhibited the attractive antimicrobial performance.

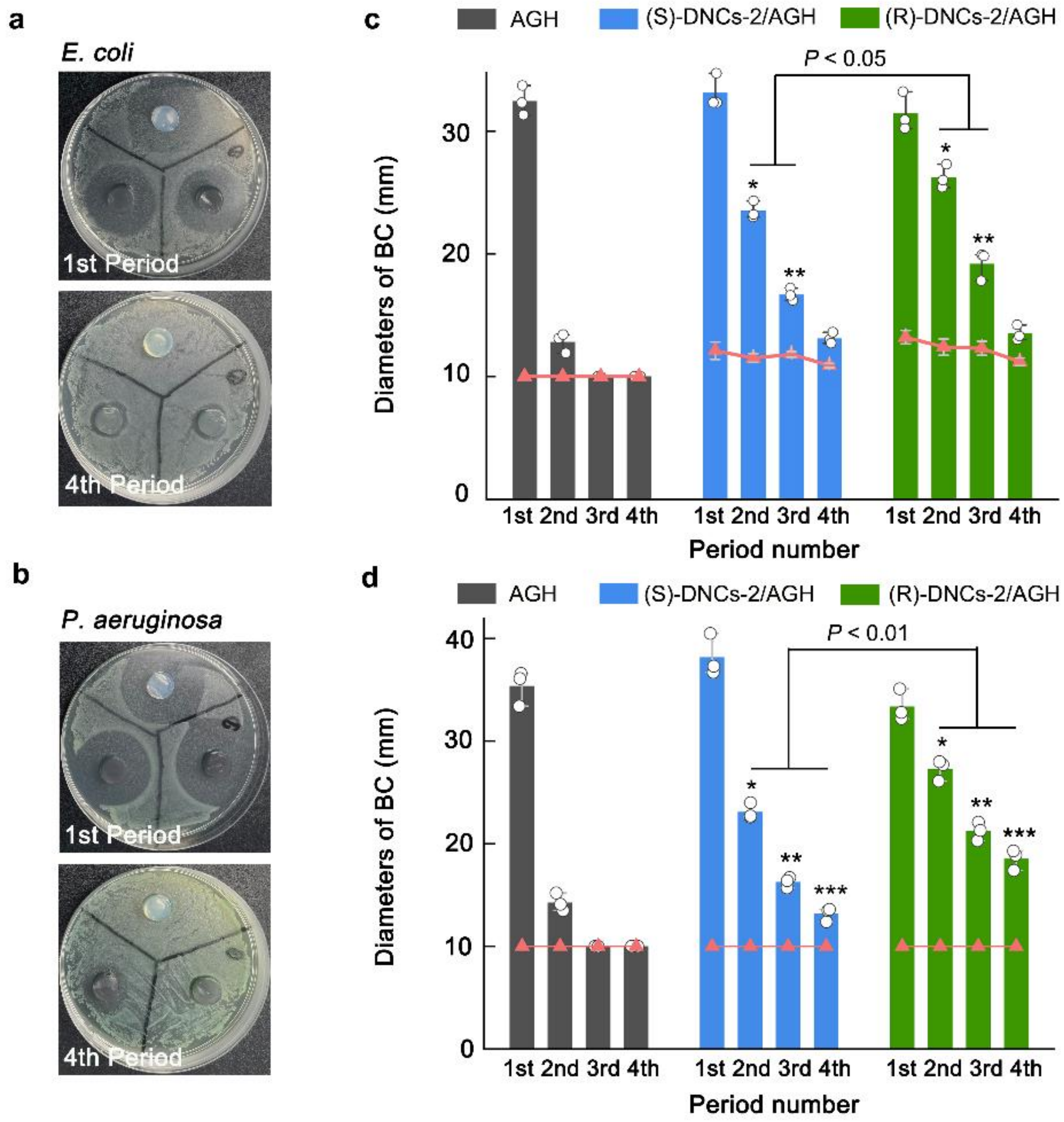

Supplementary Fig. 12 Sustained antimicrobial performances of levofloxacin-loaded composite hydrogels to *E. coli* and *P. aeruginosa*. a, **b** Pictures showing the antimicrobial performance of AGH, (R)-DNCs-2 AGH, and (S)-DNCs-2 AGH to *E. coli* and *P. aeruginosa* at the 1st and 4th period in the sustainable drug release experiment. **c**, **d** Statistic results indicating the sustainable release results through the analysis of day-dependent bacteriostatic circles of *E. coli* (**c**) and *P. aeruginosa* (**d**) in terms of diverse levofloxacin-loaded hydrogel tablets. The curves in **c, d** exhibit the bacteriostatic circles of hydrogel tablets without levofloxacin. **c** $^{*}P$=0.016, $^{**}P$=0.015, significant difference between levofloxacin-loaded (S)-DNCs-2/AGH and (R)-DNCs-2/AGH at the 2nd and 3rd period of the sustained antimicrobial experiments. **d** $^{*}P$=0.003, $^{**}P$=0.00013, $^{***}P$=0.0002, significant difference between levofloxacin-loaded (S)-DNCs-2/AGH and (R)-DNCs-2/AGH at the 2nd, 3rd, and 4th period of the sustained antimicrobial experiments. **c**, **d** Error bars represent the SD of independent experiments (n=3). Data are presented as mean values ± SD. One-sided tests were conducted and no adjustments were made for multiple comparison. Statistical analysis was performed through one-way ANOVA followed by Tukey's multiple comparison tests. Source data are provided in Source Data file.

Supplementary Note 12. Sustained antimicrobial performance of levofloxacin-loaded nanocrystals-doped hydrogels to *E. Coli* and *P. aeruginosa*

With the loading of levofloxacin, the hybrid-doped hydrogels showed enhanced efficiencies to those three kinds of bacterial. Additionally, the sustainable levofloxacin release systems worked well, indicating that the levofloxacin-loaded nanostructure-doped hydrogels can contribute to the *E. coli* and *P. aeruginosa* multi-bacterial killing systems (Supplementary Fig. 12).

Supplementary References

1. Lee, H. E. *et al.* Amino-acid- and peptide-directed synthesis of chiral plasmonic gold nanoparticles. *Nature* **556**, 360-365 (2018).
2. Li, B. L. *et al.* Principle of proximity: Plasmonic hot electrons motivate donator-adjacent semiconductor defects with enhanced electrocatalytic hydrogen evolution. *Nano Energy* **60,** 689-700 (2019).
3. Chen, X., Berner, N. C., Backes, C., Duesberg, G. S. & McDonald, A. R. Functionalization of two-dimensional MoS_2: On the reaction between MoS_2 and organic thiols. *Angew. Chem. Ed. Int.* **128**, 5897-5902 (2016).
