# Supplementary material for: Chiral nanocrystals grown from MoS2 nanosheets enable photothermally modulated enantioselective release of antimicrobial drugs
Source: Nat Commun. 2022 Nov 26;13:7289. doi: 10.1038/s41467-022-35016-8 (PMC9701227; doi:10.1038/s41467-022-35016-8)
Supplement: Supplementary file 3 — Description of Additional Supplementary Files [file 41467_2022_35016_MOESM3_ESM.pdf]

Description of Additional Supplementary Files:

Supplementary Movie 1: TEM tomography of Au/MoS2 dendritic nanocrystals synthesized in the presence of L-cysteine

Supplementary Movie 2: TEM tomography of Au/MoS2 dendritic nanocrystals synthesized in the presence of D-cysteine
